# Saved by the VAC: Minimally Invasive Removal of a Surely Fatal Right Heart Thrombus in a Patient with Advanced Heart Failure

**DOI:** 10.1155/2020/7579262

**Published:** 2020-02-18

**Authors:** James Keeton, Pedro Engel Gonzalez, Julie Cox, Robert M. Morlend, Alpesh A. Amin, Pradeep P. A. Mammen, Lynn C. Huffman, Faris G. Araj

**Affiliations:** ^1^University of Texas Health Science Center, San Antonio, Texas, USA; ^2^University of Texas Southwestern Medical Center, Dallas, Texas, USA; ^3^Baylor Scott and White Health, Dallas, Texas, USA

## Abstract

Intracardiac thrombi are associated with an increased morbidity and mortality due to their unpredictability and embolic potential. Right heart thrombus is infrequently encountered in clinical practice outside the scenario of acute pulmonary embolism with hemodynamic compromise, and even more uncommon is the presence of a massive right heart thrombus. Embolic potential is high, and historically, management has revolved around open surgical removal or systemic thrombolysis. We hereby present a case of a massive right heart thrombus in a high surgical risk patient, which was successfully removed using a percutaneous aspiration device.

## 1. Introduction

A right heart thrombus (RHT) in transit is an increasingly recognized medical emergency despite its low prevalence because it is associated with a very high mortality, especially if associated with hemodynamic instability, shock, or cardiac arrest [1, 2]. The optimal therapeutic approach is unclear given the absence of randomized control studies. However, the accepted treatment modalities include anticoagulation with systemic thrombolysis or surgical thrombectomy [2, 3]. An emerging treatment option is via catheter-based therapies, but there is still a paucity of data in this area. Critically ill patients are often at high risk of bleeding complications from thrombolytics and can have a forbiddingly high surgical risk. We hereby present a case to illustrate the complexity of treating a critically ill patient who presented with a massive RHT in transit.

## 2. Case Presentation

**Figure 1:**
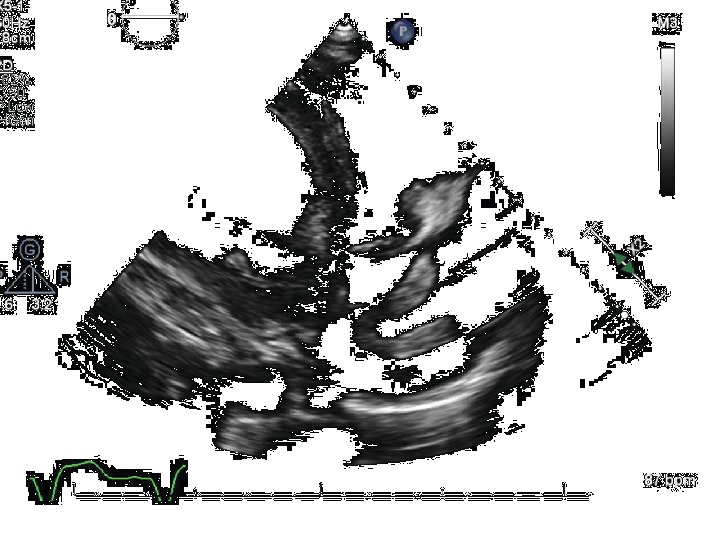
Large mobile thrombus in the right atrium prolapsing into the right ventricle.

A 61-year-old male with a history of inotrope-dependent end-stage nonischemic cardiomyopathy with a severely depressed left ventricular ejection fraction and an implantable cardioverter defibrillator (ICD) presented with bilateral lower extremity edema and seven-pound weight gain. He had no history of atrial fibrillation or venous thrombosis. This was his second hospitalization for acute on chronic decompensated systolic heart failure in a matter of a few months. His examination was notable for a heart rate of 100 bpm, a BP of 85/66 mmHg, and an oxygen saturation of 100% breathing ambient air. He was in no apparent respiratory distress. He had jugular venous distention and a third heart sound, clear lung fields, and bilateral lower extremity pitting edema. He was continued on his inotrope, and treatment with intravenous diuretic therapy was initiated. A transthoracic echocardiogram (TTE) was performed mainly to assess his right ventricular (RV) function since he was being considered for a possible orthotopic heart transplant or left ventricular assist device implant. In addition to severely depressed RV systolic function, he was found to have a new large highly mobile echo density in the right atrium intermittently prolapsing through the tricuspid valve into the right ventricle (Figure 1, [Supplementary-material supplementary-material-1]). The patient had no evidence of infectious signs or symptoms, serial blood cultures remained sterile, and the elongated serpiginous echocardiographic appearance was more suggestive of thrombus than a vegetation. Systemic anticoagulation with intravenous heparin was initiated, and a surgical consultation was obtained. Doppler ultrasound of the bilateral lower extremities did not demonstrate any deep venous thrombosis, and the TTE did not show that the thrombus was clearly attached to the lead. Testing for Factor V Leiden mutation was negative, as was testing for heparin-induced thrombocytopenia. Age-appropriate cancer screening (colon, prostate) was normal, and computed tomography of the head, chest, and abdomen/pelvis did not show any masses or pathologic lymph node enlargement. Additionally, a bone marrow biopsy was negative for any infiltrative or malignant process.

He was deemed to be too high risk for conventional surgical thrombectomy given his severe heart failure, and we were hesitant to administer systemic thrombolytic therapy given the high embolic and bleeding risk. Despite therapeutic systemic anticoagulation, a subsequent TTE 2 days later did not demonstrate reduction in the right atrial thrombus size. After discussions with the cardiothoracic surgery team and the interventional radiology team, the decision was made to attempt a minimally invasive percutaneous thrombectomy using the AngioVac Aspiration System (AngioDynamics, Latham, NY).

**Figure 2:**
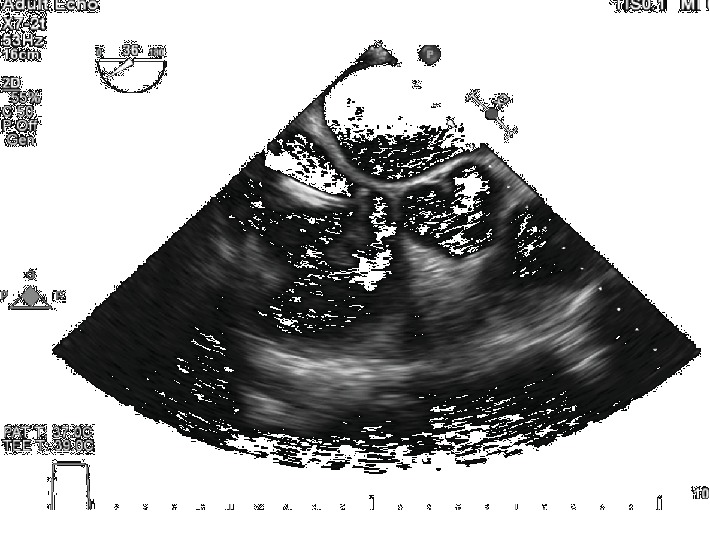
Aspiration catheter engaging the thrombus.

**Figure 3:**
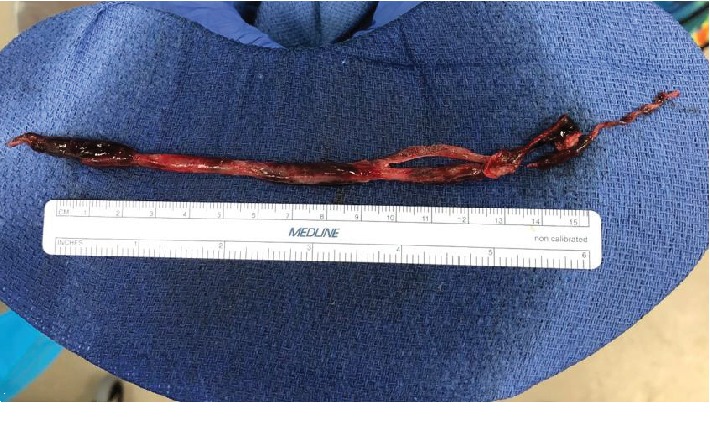
22 cm thrombus removed by the aspiration device.

Transesophageal echocardiography (TEE) was performed intraoperatively to confirm persistence of the right atrial thrombus prior to proceeding. Under general anesthesia, venous access was obtained via bilateral femoral veins to perform aspiration and reinfuse the blood. An AngioVac device was advanced to the right atrium in proximity to the thrombus (Figure 2, [Supplementary-material supplementary-material-1]). Subsequently, a 22 cm thrombus (Figure 3) was removed, and no residual thrombus was seen on intraoperative imaging. There were no apparent procedural complications, and the patient was continued on systemic intravenous anticoagulation afterwards. Subsequent cultures of the excised thrombus did not reveal any growth. The patient underwent successful cardiac transplantation three weeks later.

## 3. Discussion

The incidence of echocardiographically detected RHT in the setting of acute pulmonary embolism (PE) is low, approximating 2-4%, with the highest incidence observed in cases of acute PE with associated hemodynamic instability [1–3]. The presence of RHT in the setting of acute PE increases mortality, suggesting that detection with echocardiography may have both prognostic and therapeutic implications [1–3]. It is uncertain if the presence of a right heart thromboembolism is an independent risk factor for increased mortality, or a marker of hemodynamic dysfunction and more severe PE [3]. Without treatment, RHT in acute PE carries a 100% mortality [4, 5]. Morphologically, RHT are either elongated and serpiginous (occasionally prolapsing into the right ventricle) or either ovoid or round (typically nonmobile) [6]. Serpiginous thrombi have been associated with higher mortality, suggesting that the morphology of RHT may be a significant predictor of prognosis, although data are conflicting in this regard [3, 6].

There are no randomized clinical trial data to date to provide evidence-based guidance for any specific therapeutic modality. The main treatment options for RHT includes systemic anticoagulation, thrombolytic therapy, and either surgical or percutaneous aspiration thrombectomy. Systemic anticoagulation is considered an appropriate first line therapy for hemodynamically stable patients, although it may not be entirely effective in cases of large thrombi [3, 7]. Systemic thrombolysis can be administered rapidly and dissolves clots in several locations simultaneously (heart, pulmonary, and femoral vasculature). Systemic thrombolysis has the potential to embolize clot fragments or the entire clot by disruption of the attachment site, which can be fatal [8, 9]. Furthermore, one in five patients may suffer a bleeding complication, most feared of which would be an intracranial bleed [9]. Although small retrospective observational studies suggest that thrombolysis may be superior to anticoagulation and surgical thrombectomy, the data are conflicting [8, 9]. Open surgical thrombectomy is an invasive procedure with restrictions based on surgical risk, which leads to the exclusion of otherwise eligible candidates. Apart from the high procedural mortality [7], this involves scheduling delays and cardiopulmonary bypass.

Percutaneous aspiration thrombectomy represents a novel and less invasive method to address right heart thrombi in select patients. This treatment innovation has previously been utilized for aspiration of DVTs, including thrombi associated with inferior vena cava filters, as well as vegetations, tumors, and foreign bodies [10, 11]. The AngioVac system essentially functions as a venovenous extracorporeal membrane oxygenation circuit connected to a thrombus filter and centrifugal pump. The thrombus is aspirated, passed through a filter, and trapped in a reservoir. Then, thrombus-free blood is returned to the body [11]. Based on a single-center case series, this technique is commonly successful (up to 80%) in the removal of RHT [12]. In comparison to open surgical thrombectomy, and in the appropriately selected patient, percutaneous aspiration thrombectomy is considered a relatively safer procedure with the most common complication being access site bleeding and hematoma [12, 13]. However, there are other practical limitations to consider as well, including the device's larger profile cannula preventing use in smaller veins, rigidity of the cannula creating difficulty with maneuvering, and lack of operator experience. Additionally, patients with a contraindication to anticoagulation would not be candidates for this therapy [12, 13].

In conclusion, percutaneous aspiration thrombectomy offers a minimally invasive and potentially safer alternative to systemic thrombolytics or open surgical thrombectomy. This method successfully removed a surely fatal massive right heart thrombus in our patient with advanced heart failure, allowing him the opportunity to continue to heart transplant.

## Supplementary Materials

Supplementary Materials Movie 1: large mobile thrombus in the right atrium prolapsing into the right ventricle. Movie 2: transesophageal echocardiogram (TEE) showing thrombus and aspiration catheter.
