# Supplementary figures and images for: Saved by the VAC: Minimally Invasive Removal of a Surely Fatal Right Heart Thrombus in a Patient with Advanced Heart Failure
Source: Case Rep Cardiol. 2020 Feb 18;2020:7579262. doi: 10.1155/2020/7579262 (PMC7109588; doi:10.1155/2020/7579262)

## Slide 1
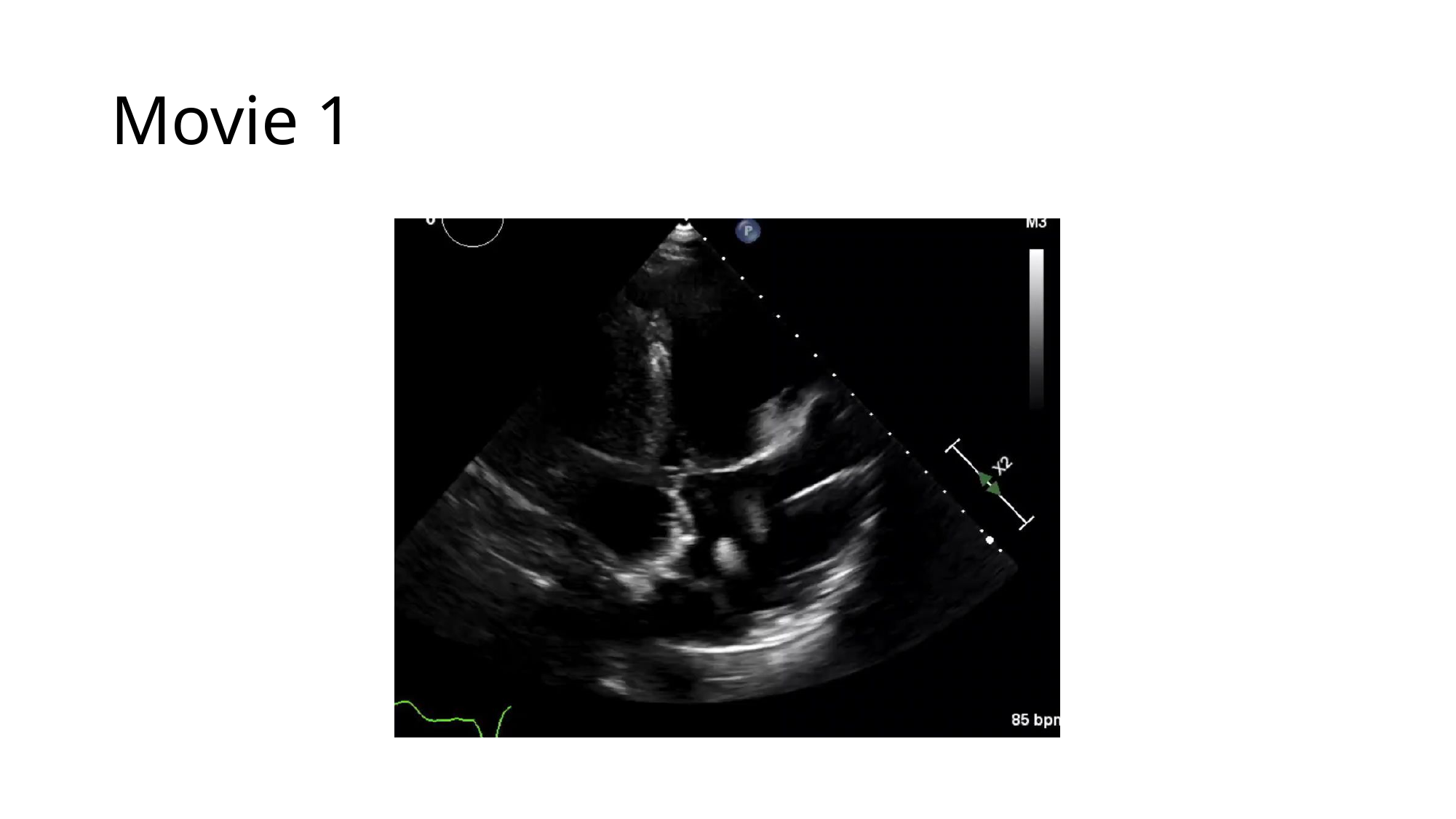

# Movie 1

## Slide 2
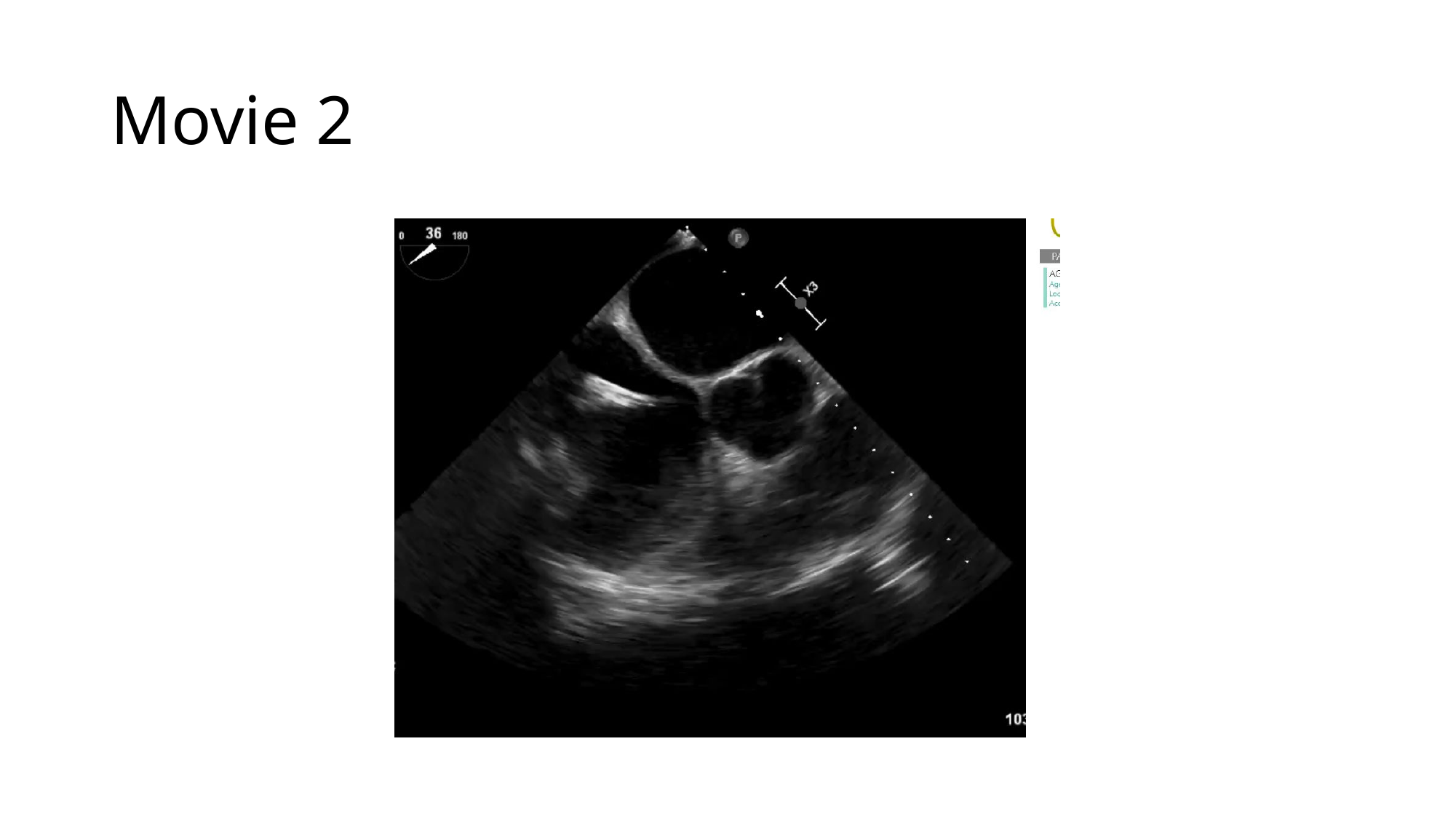

# Movie 2

Supplement: Supplementary Materials — Movie 1: large mobile thrombus in the right atrium prolapsing into the right ventricle. Movie 2: transesophageal echocardiogram (TEE) showing thrombus and aspiration catheter. [file 7579262.f1.pptx]
